# IKKβ Kinase Promotes Stemness, Migration, and Invasion in KRAS-Driven Lung Adenocarcinoma Cells

**DOI:** 10.3390/ijms21165806

**Published:** 2020-08-13

**Authors:** Felipe Silva Rodrigues, Vanessa Silva Miranda, Tatiana Correa Carneiro-Lobo, Luiza Coimbra Scalabrini, Björn Kruspig, Elena Levantini, Daniel J. Murphy, Daniela Sanchez Bassères

**Affiliations:** 1 Departamento de Bioquímica, Instituto de Química, Universidade de São Paulo, 05508-000 São Paulo, Brazil; feliperodrigues1102@gmail.com (F.S.R.); miranda.vsm@gmail.com (V.S.M.); tatty.lobo@gmail.com (T.C.C.-L.); scalabrini.luiza@gmail.com (L.C.S.); 2 Institute of Cancer Sciences, University of Glasgow, Glasgow G61 1QH, UK; bjorn.kruspig@glasgow.ac.uk (B.K.); daniel.murphy@glasgow.ac.uk (D.J.M.); 3 Beth Israel Deaconess Medical Center, Harvard Medical School, Boston, MA 02115, USA; elevanti@bidmc.harvard.edu; 4 Istituto di Tecnologie Biomediche, Consiglio Nazionale dele Ricerche, 56124 Pisa, Italy; 5 Cancer Research UK Beatson Institute, Glasgow G61 1BD, UK

**Keywords:** KRAS, lung cancer, IKKβ kinase, stemness, cancer stem cells, NF-κB signalling, migration, invasion

## Abstract

KRAS oncogenic mutations are widespread in lung cancer and, because direct targeting of KRAS has proven to be challenging, KRAS-driven cancers lack effective therapies. One alternative strategy for developing KRAS targeted therapies is to identify downstream targets involved in promoting important malignant features, such as the acquisition of a cancer stem-like and metastatic phenotype. Based on previous studies showing that KRAS activates nuclear factor kappa-B (NF-κB) through inhibitor of nuclear factor kappa-B kinase β (IKKβ) to promote lung tumourigenesis, we hypothesized that inhibition of IKKβ would reduce stemness, migration and invasion of KRAS-mutant human lung cancer cells. We show that KRAS-driven lung tumoursphere-derived cells exhibit stemness features and increased IKKβ kinase activity. IKKβ targeting by different approaches reduces the expression of stemness-associated genes, tumoursphere formation, and self-renewal, and preferentially impairs the proliferation of KRAS-driven lung tumoursphere-derived cells. Moreover, we show that IKKβ targeting reduces tumour cell migration and invasion, potentially by regulating both expression and activity of matrix metalloproteinase 2 (MMP2). In conclusion, our results indicate that IKKβ is an important mediator of KRAS-induced stemness and invasive features in lung cancer, and, therefore, might constitute a promising strategy to lower recurrence rates, reduce metastatic dissemination, and improve survival of lung cancer patients with KRAS-driven disease.

## 1. Introduction

Lung cancer is the main cause of cancer-related deaths in the world, and despite the increasing advances in the development of new, targeted therapies, the 5-year survival rates remain lower than 20%. The most common genetic alterations found in lung cancer patients include activating-point mutations in *KRAS*, which are present in a third of lung adenocarcinoma patients, and are causally correlated with poor prognosis [1]. Although there has been promising recent progress in the development of inhibitors capable to selectively target KRAS^G12C^ mutants [2,3,4], direct inhibition of KRAS has proven to be remarkably challenging to date [5]. On the other hand, targeting traditional downstream effectors of KRAS has only reached limited efficacy due to poor therapeutic windows coupled with paradoxical pathway activation by signalling loops [5]. In order to overcome these challenges, identification and characterization of new druggable targets in the KRAS-induced signalling pathways that promote important malignant features is urgently warranted for the development of novel and more effective therapeutic strategies for lung cancer, as well as other RAS-driven malignancies.

One very important malignant feature of many tumours that has important therapeutic implications is the existence of malignant cells with stemness properties. These stem-like tumour-initiating cells (TICs) are able to self-renew and sustain tumour growth and are considered to be one of the main explanations for tumour resistance, recurrence, and metastasis [6]. Moreover, purification and/or enrichment of these phenotypically distinct cancer cells from various tumours, including those of the lung [7], results in a subpopulation of tumour cells with an exceptional tumourigenic capacity when injected into immunocompromised mice. These cells also display enhanced resistance to chemotherapeutic drugs, and increased invasive and metastatic capacity [8]. In this regard, oncogenic KRAS not only drives cancer cell proliferation, but it has been implicated in metastasis formation [9,10,11] and in driving the cancer stem-like phenotype [12,13,14,15,16].

One important mechanism driving lung tumourigenesis is the KRAS-mediated activation of the nuclear factor kappa-B (NF-κB) transcription factor. NF-κB has been shown to promote KRAS-induced lung tumour growth, proliferation and survival [17,18]. Interestingly, NF-κB has also been implicated in promoting metastasis in different tumour models [19] and has been extensively implicated in regulating stemness behaviour of TICs in various tumours [20].

A potential druggable target in the KRAS-induced NF-κB activation pathway is the inhibitor of nuclear factor kappa-B kinase β (IKKβ). IKKβ is a catalytic subunit of the inhibitor of nuclear factor kappa-B kinase (IKK) complex, which also includes a second catalytic subunit (IKKα) and a regulatory subunit (IKKγ). The IKK complex mediates canonical NF-κB activation by phosphorylating and thereby promoting the proteasome-mediated degradation of the inhibitory protein inhibitor of nuclear factor kappa-B alpha (IκBα), which sequesters NF-κB in the cytoplasm [19]. Not surprisingly, NF-κB activation by oncogenic KRAS in the lung involves the canonical pathway [21] and requires IKKβ [22].

Based on this evidence we hypothesized that IKKβ would promote KRAS-driven lung cancer stemness and invasion. In support of this hypothesis, genetic or systemic IKKβ inhibition not only reduces KRAS-induced lung tumour growth by reducing cell proliferation [22,23], but also reduces KRAS-induced angiogenesis [24], a cancer hallmark that is associated with poor prognosis and contributes to the process of metastasis [25].

Here, using an established tumoursphere formation assay [15,26] to enrich for lung TICs, we show that IKKβ is critical to sustain stemness-associated features in KRAS-driven lung cancer cells. Using pharmacological and genetic approaches, we demonstrate that IKKβ contributes to the expression of stemness-associated genes, tumoursphere formation and self-renewal. Remarkably, targeting of IKKβ activity preferentially reduces the proliferation of TICs. Furthermore, IKKβ targeting also decreases tumour cell migration, invasion and expression of metastasis-associated genes. Finally, our data suggest that IKKβ kinase inhibition therapy may clinically benefit KRAS-driven lung cancer patients by depleting the pool of stem-like TICs, thereby decreasing the risk of tumour recurrence and metastasis.

## 2. Results

### 2.1. KRAS-Mutant Lung Tumoursphere-Derived Cells Exhibit Stemness Features and Increased IKKβ Kinase Activity

**Figure 1:**
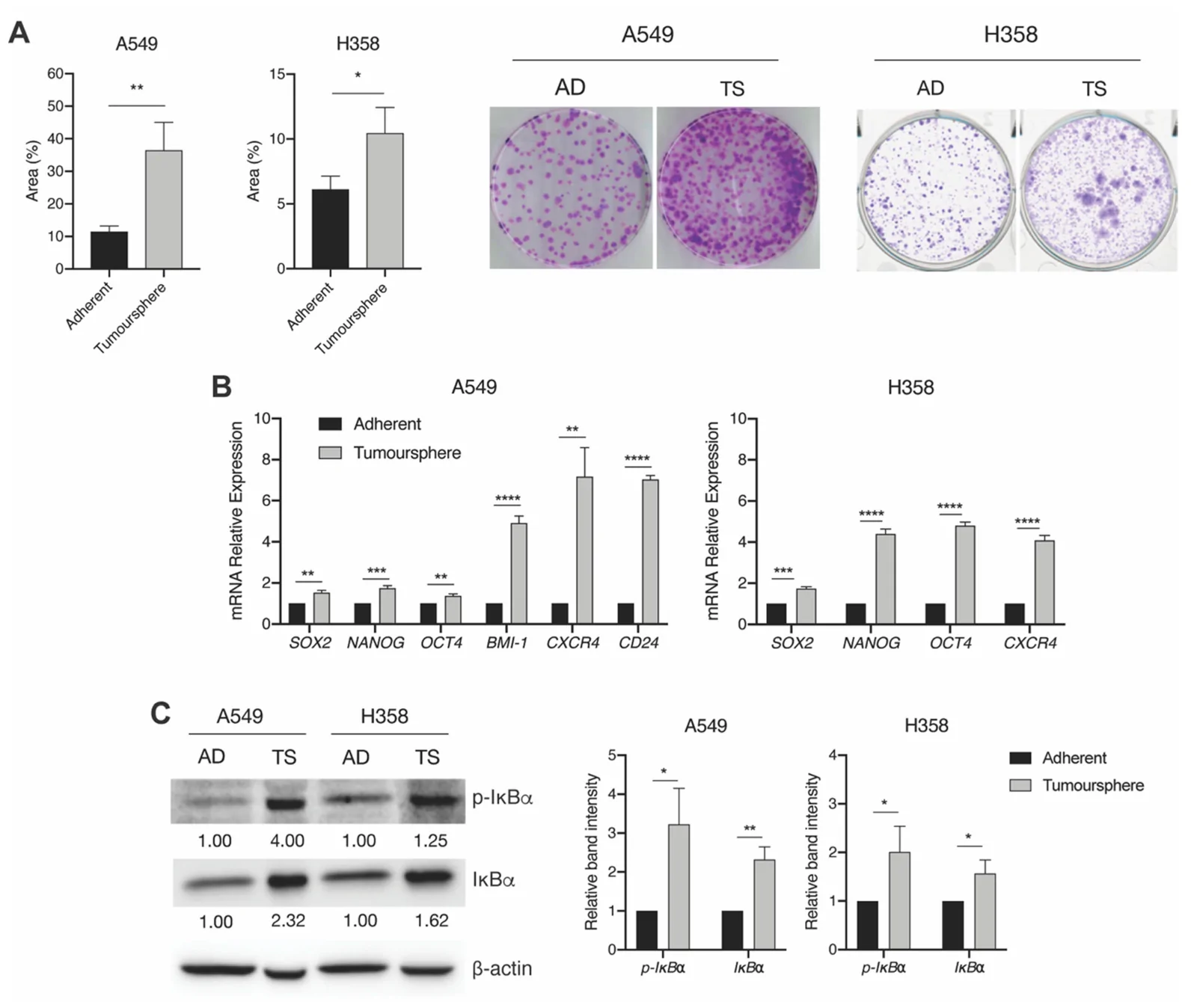
KRAS-mutant tumoursphere-derived cells exhibit stemness features and increased IKKβ kinase activity. (**A**) Clonogenic assays of adherent (AD) and tumoursphere-derived (TS) A549 and H358 cells. Cells were plated and colonies formed were stained with crystal violet and colony area was analysed using Image J software. Images shown are representative of three independent experiments. (**B**) Relative expression of *SOX2*, *OCT4*, *NANOG*, *CXCR4*, *BMI1,* and *CD24* was analysed by real-time quantitative PCR in adherent (AD) and tumoursphere-derived (TS) A549 and H358 cells using *β-ACTIN* as endogenous control. (**C**) Western blotting of adherent (AD) and tumoursphere-derived (TS) A549 and H358 cells. Antibodies used are indicated. Protein bands were quantitated and normalized to the reference sample using ImageJ software. Nitrocellulose membrane was cut before probing with the respective primary antibody and full membrane blots are presented in Figure S1. Images shown are representative of three independent experiments. In all cases, bar graphs represent average ±1 SD of three independent experiments (n = 3). Statistical significance was determined by Student’s *t*-test (* *p* < 0.05, ** *p* < 0.01, *** *p* < 0.001, **** *p* < 0.0001). Groups being compared are indicated by horizontal bars.

Because IKKβ targeting in KRAS-induced lung cancer reduces tumour histological grade, angiogenesis and is required for activation of NF-κB [17,22,24], which, in turn, has been shown to be critical for TIC activity in different contexts [20], we hypothesised that IKKβ would be activated in KRAS-induced lung TICs. In order to test this hypothesis, we used a well-established tumoursphere culture system to enrich for TICs [15,26]. As expected, when compared to adherent cultures, KRAS-mutant A549 or H358 tumoursphere-derived cells were more clonogenic (Figure 1A) and expressed significantly elevated levels of stemness-related genes SRY (sex determining region Y)-box 2 (*SOX2)*, *NANOG*, octamer-binding transcription factor 4 (*OCT4),* and C-X-C chemokine receptor type 4 (*CXCR-4)* (Figure 1B). A549 tumourspheres also had increased expression of B cell-specific Moloney murine leukaemia virus integration site 1 (*BMI1)* and the stem cell surface marker cluster of differentiation (CD) 24. Remarkably, when compared to adherent cells, both A549 and H358 tumourspheres displayed a 3.2-fold and a 2.0-fold increase in phosphorylation of the IKKβ substrate IκBα respectively (Figure 1C and Figure S1). Total IκBα, which is an NF-κB-activated gene, was also increased by 2.32-fold and 1.62-fold, respectively, indicating increased activity of the IKK/NF-κB pathway in KRAS-positive lung tumourspheres enriched for TIC activity.

### 2.2. IKKβ Targeting in KRAS-Positive Lung Cancer Cells Reduces the Expression of Stemness-Associated Genes

**Figure 2:**
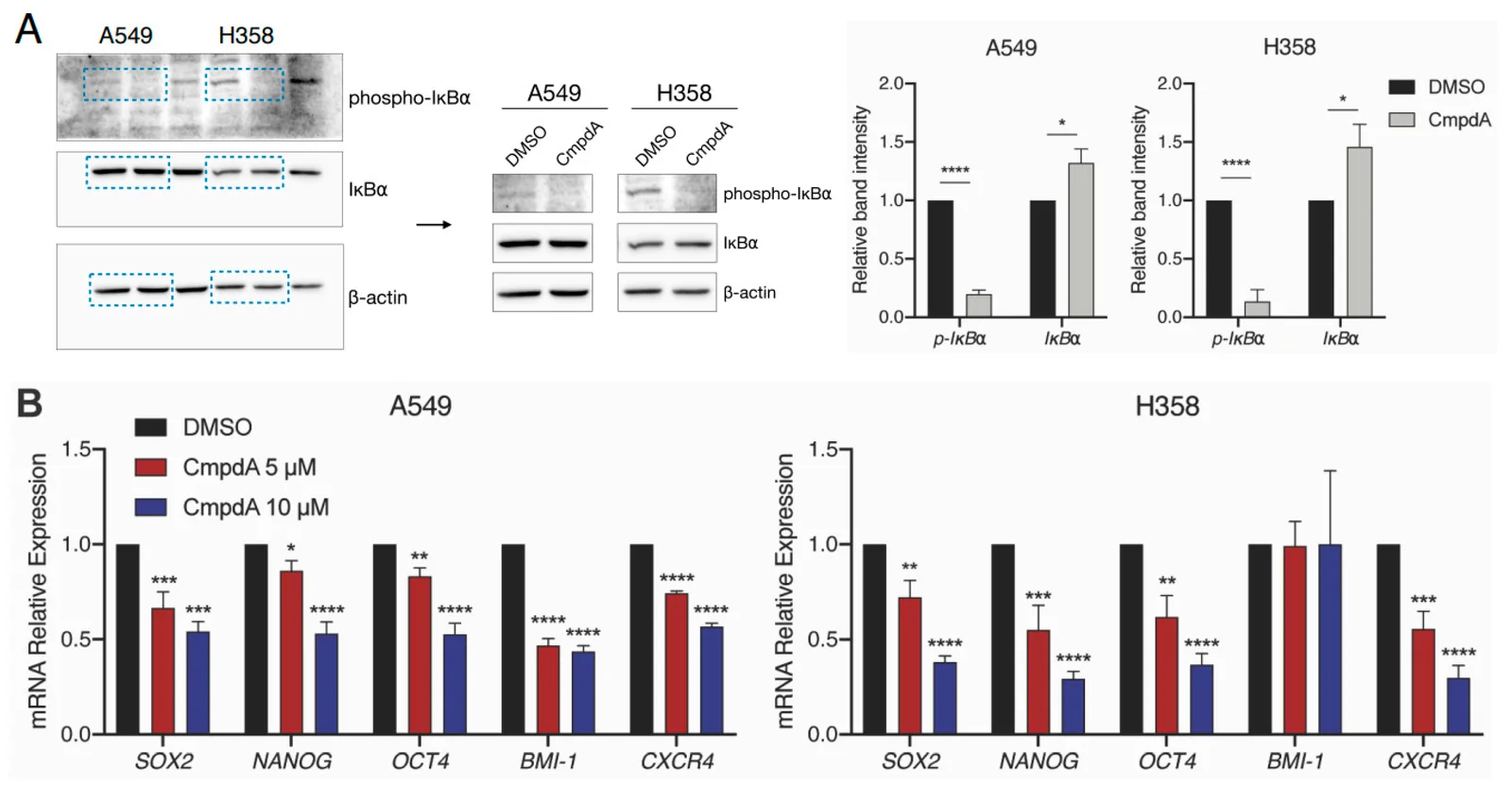
IKKβ targeting reduces the expression of stemness-associated genes in KRAS-mutant lung cancer cells. (**A**) Western blotting of A549 and H358 cells treated with 0.1% DMSO or 5 µM Compound A (CmpdA) for 30 min. Antibodies were used as indicated. Nitrocellulose membrane was cut before probing with the respective primary antibody, and full membrane blots are presented in Figure S2. The representative Western blots are shown. The protein bands were quantitated and normalized to the reference samples (0.1% DMSO-treated samples) using Image J software (version 1.51). (**B**) A549 and H358 cells were treated with 0.1% DMSO or the indicated concentrations of CmpdA for 48 h, and expression of *SOX2*, *OCT4*, *NANOG*, *BMI1*, and *CXCR4* was evaluated by qRT-PCR using *β-ACTIN* as an endogenous control. The bar graphs represent average ±1 SD of three independent experiments (n = 3). Statistical significance was determined by one-way ANOVA with the post hoc Turkey test. (* *p* < 0.5, ** *p* < 0.01, *** *p* < 0.001, **** *p* < 0.0001) by comparing the CmpdA-treated groups with the DMSO-treated group.

Next, we targeted IKKβ with Compound A (CmpdA), a highly selective IKKβ inhibitor [27]. As expected, CmpdA treatment reduced IκBα phosphorylation consistent with inhibition of IKKβ activity (Figure 2A and Figure S2). Interestingly, looking at expression of a panel of stemness-related genes, we found that IKKβ inhibition in KRAS-positive A549 cells significantly reduced expression of stem cell transcription factors SOX2, NANOG, OCT4, and BMI1, as well as the TIC surface marker CXCR-4 (Figure 2B, left panel). With the exception of BMI1, identical results were obtained by CmpdA-mediated IKKβ targeting in KRAS-mutant H358 cells (Figure 2B, right panel).

### 2.3. IKKβ Targeting Reduces Tumoursphere Formation, Self-Renewal, and Preferentially Impairs Proliferation of TIC-Enriched KRAS-Mutant Cells

**Figure 3:**
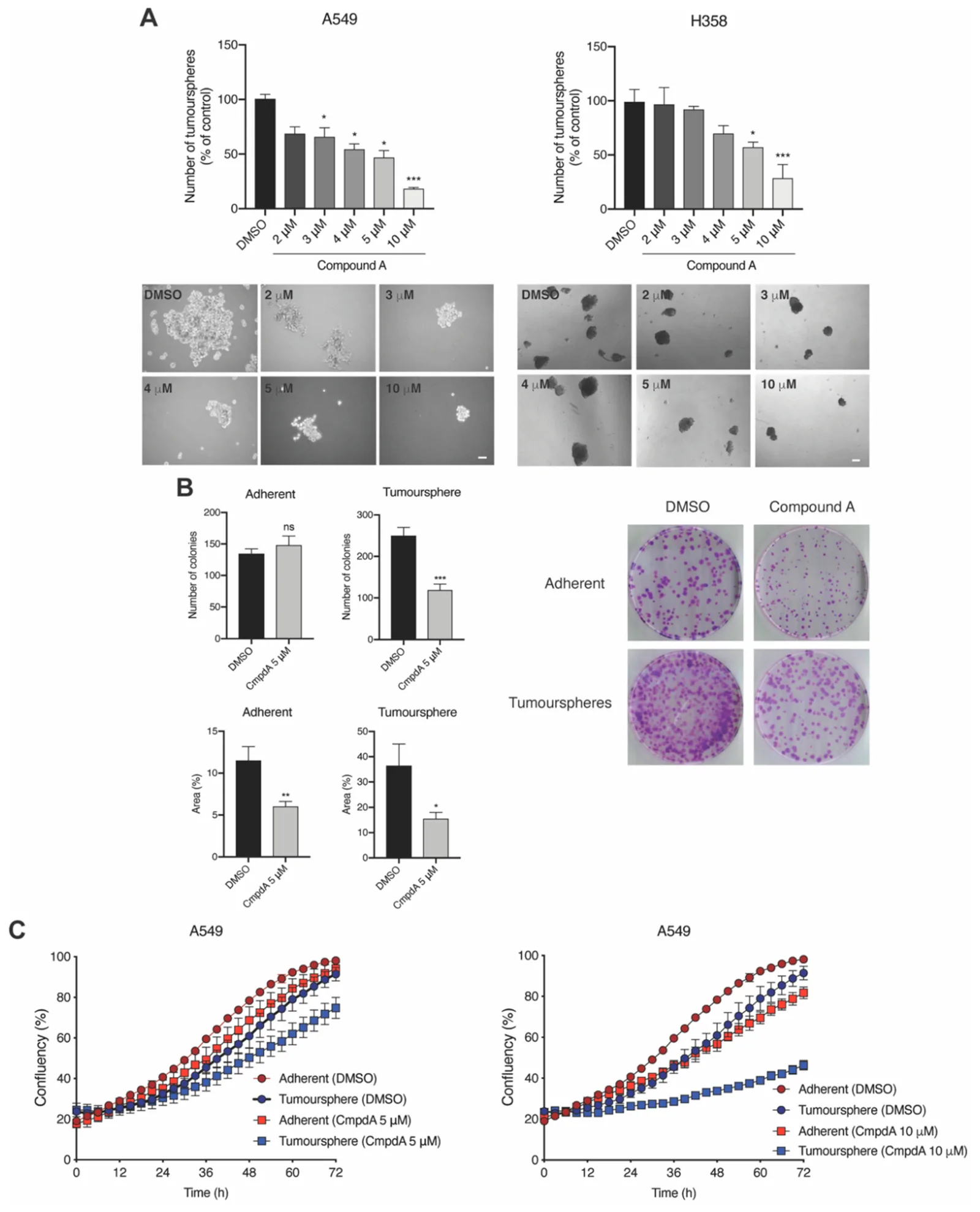
IKKβ targeting with CmpdA reduces tumoursphere formation and preferentially impairs proliferation of KRAS-mutant tumoursphere-derived cells. (**A**) A549 and H358 cells were treated with the indicated concentrations of CmpdA or with vehicle control (0.1% DMSO) and plated for tumoursphere cultures. Tumoursphere number was determined by manual counting. Images shown are representative of three independent experiments (n = 3). White scale bars represent 50 µm. (**B**) Clonogenic assays of A549 adherent cells treated with CmpdA or vehicle control (0.1% DMSO) were compared to clonogenic assays of A549 cells derived from CmpdA-treated or control-treated (0.1% DMSO) tumourspheres. Adherent or tumoursphere-derived cells were plated and colonies formed were stained with crystal violet. Colony number and colony area were quantified using Image J software. Images shown are representative of three independent experiments (n = 3). (**C**) Growth curves measured by IncuCyte time-lapse video microscopy of adherent-derived and tumoursphere-derived A549 cells upon treatment with 0.1% DMSO and 5 µM CmpdA (left) or 10 µM CmpdA (right). A representative growth curve of two independent experiments (n = 2) is shown for each condition. Error bars show ±1 SD for technical triplicates. In all cases, bar graphs represent average ± 1 SD of three independent experiments (n = 3). Statistical significance was determined by one-way ANOVA with a post hoc Turkey test (* *p* < 0.5, *** *p* < 0.001) (**A**) or by Student’s *t*-test (* *p* < 0.05, ** *p* < 0.01, *** *p* < 0.001, ns = not significant) (**B**), by comparing CmpdA-treated groups with the DMSO-treated group.

After determining that IKKβ promotes the expression of stemness-related genes, we sought out to investigate how IKKβ would affect TIC function. For that purpose, we treated A549 and H358 cells with CmpdA and analysed tumoursphere formation and TIC proliferation. We found that CmpdA reduced the ability of A549 and H358 cells to form tumourspheres in a dose-dependent manner (Figure 3A). This reduction in tumoursphere formation is not caused by cell death, as CmpdA treatment did not significantly increase A549 or H358 cell death (Figure S3A). Moreover, whereas CmpdA treatment did not affect the ability of adherent A549 cells to form colonies, it decreased colony formation of A549 tumoursphere-derived TIC-enriched cells by half (Figure 3B). It is noteworthy that, even though CmpdA did not affect the number of colonies formed by adherent A549 cells, it did affect colony size (Figure 3B). This can be explained by the fact CmpdA leads to a dose-dependent reduction in A549 proliferation (Figure S3B). In accordance, whereas CmpdA treatment of adherent A549 cells led to a dose-dependent decrease in proliferation, resulting, after 72 h, in a 4% reduction at 5 µM CmpdA and a 16% reduction at 10 µM CmpdA respectively (Figure 3C), CmpdA treatment of A549 tumoursphere-derived cells led to a much more pronounced decrease in proliferation, resulting, after 72 h, in a 16% reduction at 5 µM CmpdA and a 45% reduction at 10 µM CmpdA respectively (Figure 3C). These results indicate that IKKβ targeting preferentially impairs proliferation of TIC-enriched KRAS-mutant cells.

**Figure 4:**
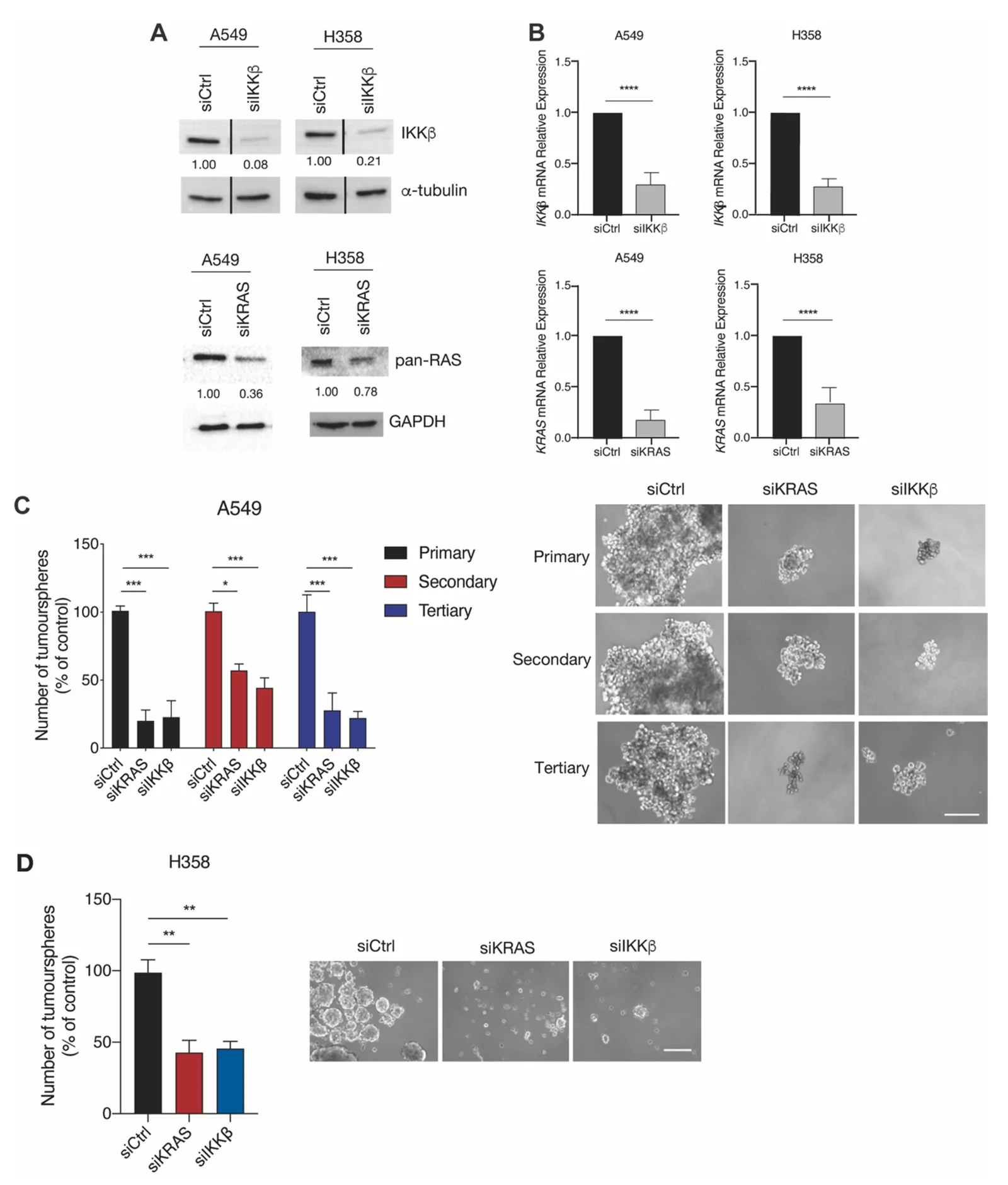
Small interfering RNA (siRNA)-mediated IKKβ targeting reduces tumoursphere formation and self-renewal of KRAS-mutant lung cancer cells. A549 and H358 cells were transfected with a non-targeting control siRNA (siCtrl) or with siRNA SMARTpools targeting KRAS (siKRAS) or IKKβ (siIKKβ) as described in methods. (**A**) Protein lysates were collected 96 h post-transfection and evaluated by Western Blotting with the indicated antibodies. Protein bands were quantitated and normalized to the reference samples (siCtrl samples). Nitrocellulose membrane was cut before probing with the respective primary antibody. Lanes from blots cropped from different membranes are separated by a black line and full membrane blots are presented in Figure S4. Images shown are representative of three independent experiments (n = 3). (**B**) Expression of *KRAS* (left panel) or *IKKβ* (right panel) was analysed 72 h post-transfection by RT-qPCR in each cell line as indicated using *GAPDH* as endogenous control. (**C**) Serial tumoursphere formation assay of siKRAS- or siIKKβ-transfected A549 cells compared to siCtrl-transfected A549 cells. Representative images of primary, secondary and tertiary A549 tumourspheres for each siRNA transfection condition are shown. White scale bar represents 100 µm. (**D**) Primary tumoursphere formation assay of siKRAS- or siIKKβ-transfected H358 cells compared to siCtrl-transfected H358 cells. Representative images of H358 tumourspheres for each siRNA transfection condition are shown. White scale bar represents 100 µm. In all cases, bar graphs represent average ±1 SD of three independent experiments (n = 3). Statistical significance was determined by the Student’s *t*-test (**** *p* < 0.0001) by comparing siCtrl-transfected groups with siKRAS- or with siIKKβ-transfected groups (B) or by one-way ANOVA with a post hoc Turkey test (* *p* < 0.5, ** *p* < 0.01, *** *p* < 0.001) (**C** and **D**). Groups being compared are indicated by horizontal bars.

In order to evaluate the impact of IKKβ targeting on TIC self-renewal, we targeted IKKβ in A549 and H358 cells by small interfering RNA (siRNA)-mediated transfection and evaluated the ability of targeted cells to form tumourspheres in serial passages. As can be seen in Figure 4A, IKKβ or KRAS targeting by siRNA-mediated transfection resulted in a 92% and 79% reduction in IKKβ protein expression and a 64% and a 22% in reduction in RAS protein expression in A549 and H358 cells, respectively (Figure 4A and Figure S4). This reduction in protein expression was associated with a 70% and 63% reduction in IKKβ mRNA expression, and an 81% and a 71% in reduction in KRAS mRNA expression in A549 and H358 cells, respectively (Figure 4B). The lower reduction of KRAS observed at the protein level compared to the mRNA level stems from the fact that the antibody used to detect KRAS also detects the NRAS and HRAS isoforms, which are not targeted by the siRNA used. Taken together, these results indicate that our siRNA targeting approach was successful.

Interestingly, IKKβ targeting by RNA interference decreased the ability of A549 cells to form primary tumourspheres by 80%, secondary tumourspheres by 60% and tertiary tumourspheres by 80%. These results were recapitulated by targeting KRAS, which also resulted in a similar reduction in primary (80%), secondary (40%) and tertiary (70%) tumoursphere formation (Figure 4C). Even though serial tumoursphere passage analysis was not feasible in H358 cells, due to the fact that an insufficient number of cells were available for passage into secondary cultures when IKKβ or KRAS was targeted, siRNA-mediated IKKβ or KRAS targeting also significantly reduced primary tumoursphere formation (by approximately 60% for both IKKβ and KRAS, Figure 4D). Interestingly, whereas H358 cells have been shown to be sensitive to KRAS inhibition, A549 cells do not lose viability upon KRAS inhibition and both are resistant to IKKβ inhibition under adherent conditions [22]. Therefore, even though we cannot rule out the possibility that the reduction in primary tumoursphere formation in H358 cells upon KRAS silencing may reflect a loss of intrinsic viability, the reduction observed in A549 cells and upon IKKβ inhibition cannot be attributed to an intrinsic cell viability loss. Therefore, taken together, these results indicate that the KRAS/IKKβ pathway promotes lung tumoursphere formation and self-renewal, thereby implicating that targeting this pathway may affect TIC function.

### 2.4. IKKβ Kinase Targeting Reduces KRAS-Mutant Lung Cell Migration and Invasion

**Figure 5:**
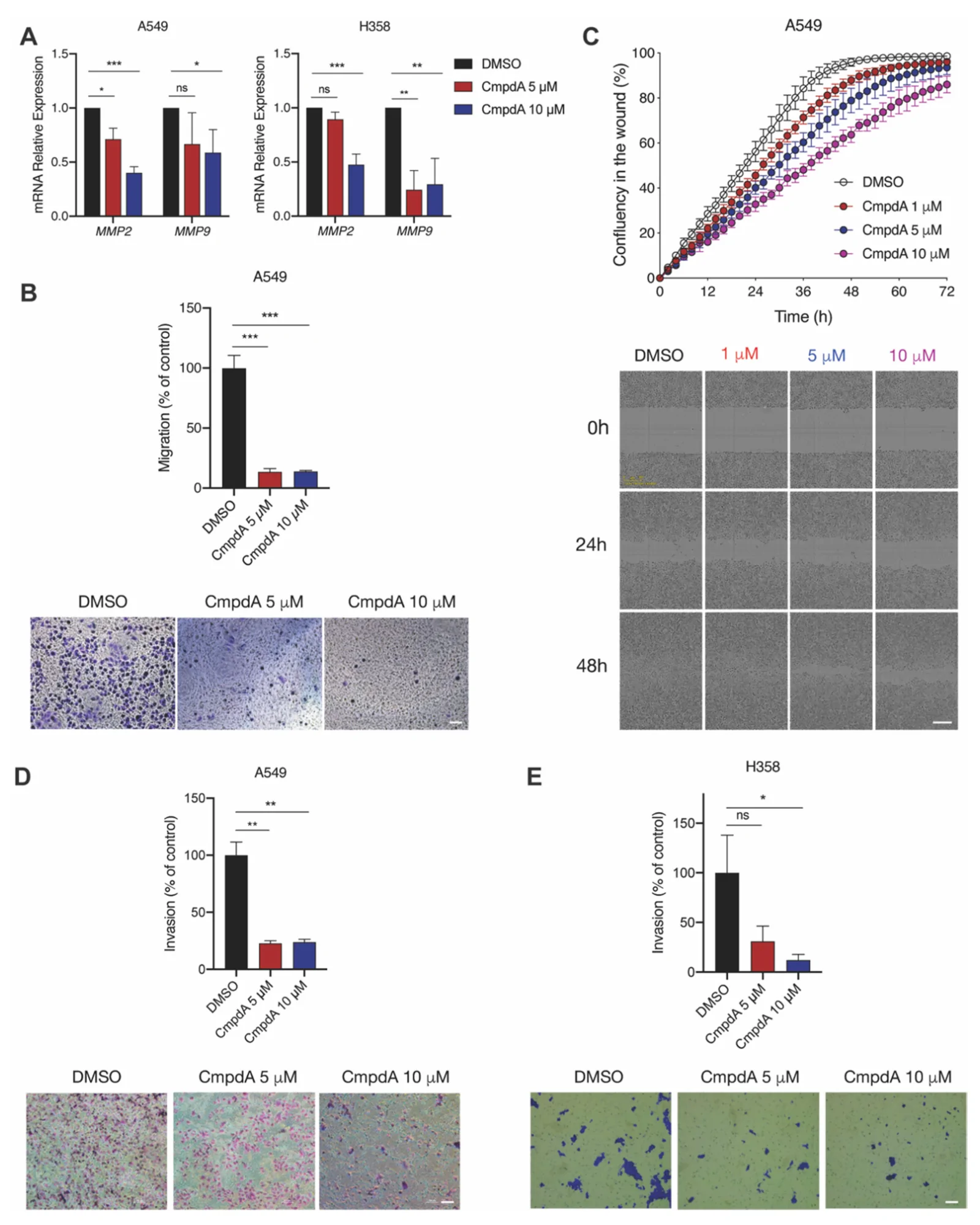
IKKβ kinase targeting with Compound A reduces the expression of matrix metalloproteinase genes, tumour cell migration and invasion. A549 and H358 cells were treated with 0.1% DMSO or the indicated concentrations of CmpdA. (**A**) After 24 h, expression of *MMP2* and *MMP9* was evaluated by RT-qPCR using *β-ACTIN* as endogenous control. (**B**) After 24 h, transwell migration assays were performed as described in methods. Images shown are representative of three independent experiments (n = 3). White scale bar represents 100 µm. (**C**) Real-time wound healing assays of A549 cells were performed by IncuCyte time-lapse video microscopy over 72 h of treatment with 0.1% DMSO or the indicated concentrations of CmpdA. Results are expressed as percentage of confluence in the wound. A representative wound confluence curve of two independent experiments is shown for each condition. Error bars show ±1 SD for technical triplicates. Representative images of A549 wound-healing assays at 0, 24 and 48 h are shown. White scale bar represents 300 µm. (**D**) After 24 h of treatment of A549 and (**E**) H358 cells with 0.1% DMSO or the indicated concentrations of CmpdA, transwell invasion assays were performed as described in methods. Images shown are representative of three independent experiments (n = 3). White scale bars represent 100 µm. In all cases, bar graphs represent average ±1 SD of three independent experiments (n = 3). Statistical significance was determined by one-way ANOVA with a post hoc Turkey test. (* *p* < 0.5, ** *p* < 0.01, *** *p* < 0.001, ns = not significant). Groups being compared are indicated by horizontal bars.

In addition to the ability to self-renew, TICs are thought to be responsible for metastatic dissemination and TICs have been shown to be intrinsically migratory and invasive [28]. Because IKKβ promotes TIC function and because it activates the NF-κB pathway, which has also been implicated in promoting metastasis in different tumour models [29], we hypothesized that it would also promote KRAS-induced migration and invasion. Intriguingly, we found that IKKβ targeting by CmpdA in A549 and H358 cells reduces expression of matrix metalloproteinases 2 (MMP2) and 9 (MMP9) (Figure 5A), which are involved in promoting cell invasion [30]. Based on this result, we evaluated the effect of IKKβ targeting with CmpdA on cell migration and invasion. Although H358 cells did not migrate in transwell and wound-healing assays, even in control conditions, CmpdA treatment significantly reduced A549 cell transwell migration independent of CmpdA dose, as an 85% reduction was observed with 5 and 10 µM of CmpdA (Figure 5B). In accordance with this result, CmpdA treatment also reduced A549 wound-healing migration (Figure 5C). This reduction was more pronounced 48 h after CmpdA administration, where a 16% reduction was observed with 5 µM CmpdA and a 30% reduction with 10 µM CmpdA (Figure 5C). Moreover, IKKβ targeting with CmpdA reduced A549 invasion (Figure 5D) in a dose-independent manner, as a reduction of 82% and 83% were observed with 5 and 10 µM CmpdA, respectively. Interestingly, even though H358 cells did not display a migratory phenotype, they were invasive, which suggests that signalling by matrix proteins present in Matrigel is crucial to activate H358 migration. Similarly to what we observed with A549 cells, IKKβ targeting with CmpdA in H358 cells also reduced cell invasion, but in a dose-dependent manner, as a 70% and 90% reduction was observed with 5 µM and 10 µM CmpdA, respectively (Figure 5E).

**Figure 6:**
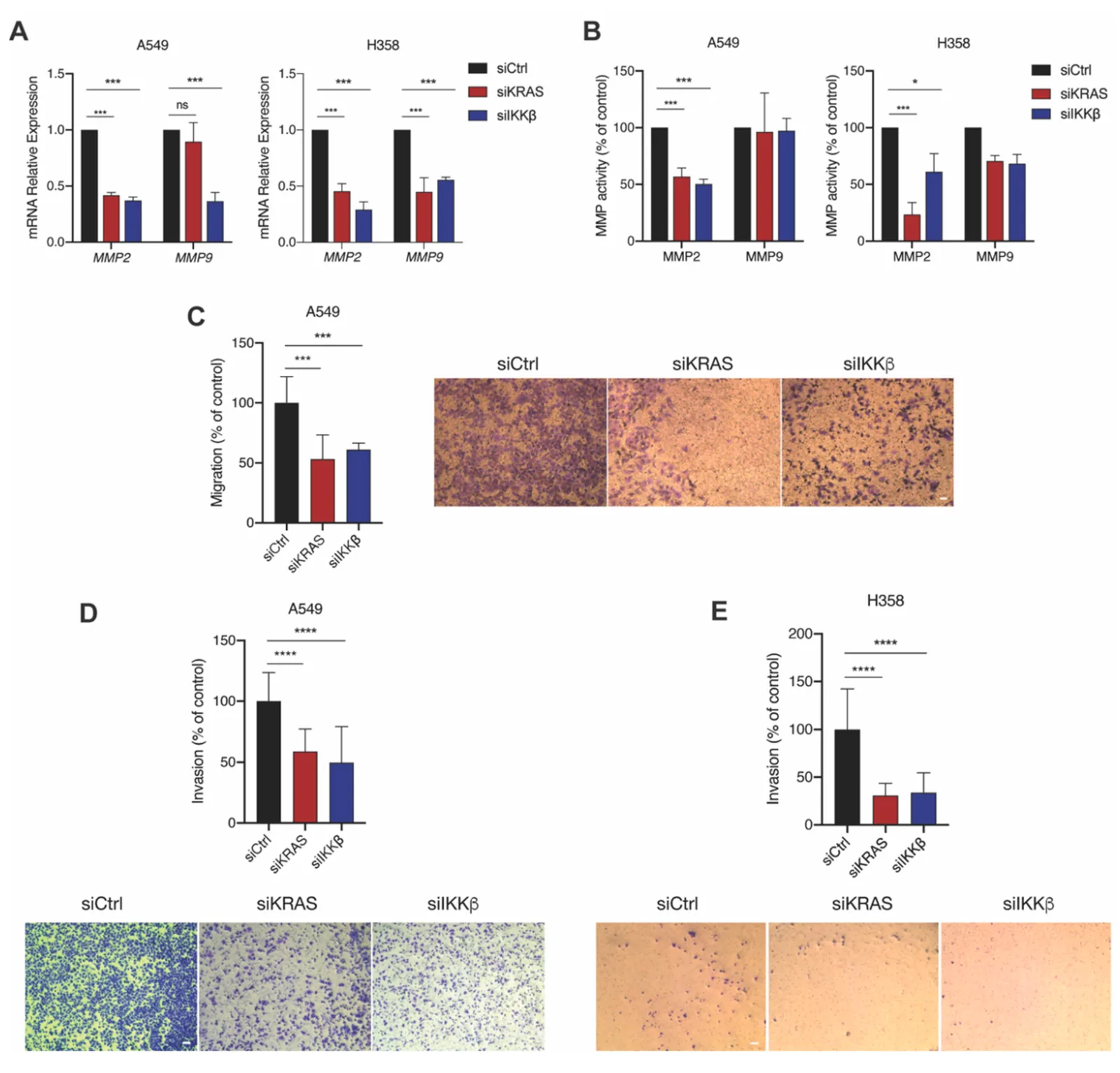
siRNA-mediated targeting of IKKβ or KRAS reduces the expression and activity of matrix metalloproteinases, tumour cell migration and invasion. A549 and H358 cells were transfected with a non-targeting control siRNA (siCtrl) or with siRNA SMARTpools targeting KRAS (siKRAS) or IKKβ (siIKKβ) as described in methods. (**A**) After 72 h, expression of *MMP2* and *MMP9* was evaluated by RT-qPCR using *GAPDH* as endogenous control. (**B**) Conditioned culture medium was collected 96 h post-transfection and matrix metalloproteinase 2 (MMP2) and matrix metalloproteinase 9 (MMP9) activity was determined using ELISA-based Biotrack Activity Assay Systems (GE Healthcare). (**C**) Transwell cell migration assays were performed as described in methods 96 h post-transfection. Images shown are representative of three independent experiments (n = 3). White scale bar represents 50 µm. (**D**) Transwell cell invasion assays for A549 and (**E**) H358 were performed as described in methods 72 h post-transfection. Images shown are representative of three independent experiments (n = 3). White scale bars represent 50 µm. In all cases, bar graphs represent average ±1 SD of three independent experiments (n = 3). Statistical significance was determined by one-way ANOVA with a post hoc Turkey test (* *p* < 0.05, *** *p* < 0.001, **** *p* < 0.0001). Groups being compared are indicated by horizontal bars.

These results were further corroborated by IKKβ targeting by RNA interference. siRNA-mediated IKKβ inhibition in both A549 and H358 cells reduced expression of MMP2 by 60% and 70% and MMP9 by 75% and 55%, respectively (Figure 6A). siRNA-mediated KRAS targeting led to similar reductions in MMP2 expression in both cell lines (Figure 6A). Moreover, even though KRAS targeting did not affect MMP9 expression in A549 cells, it reduced MMP9 expression by 55% in H358 cells (Figure 6A). Next, we assessed if this reduced expression would be associated with reduced MMP activity. We found that MMP2 activity is similarly reduced by IKKβ or KRAS targeting in both A549 and H358 cells (Figure 6B), whereas IKKβ or KRAS targeting did not affect MMP9 activity (Figure 6B). Even though some differences in MMP expression and activity upon IKKβ targeting were observed between cells lines, all cell lines displayed reduced expression and/or activity of at least one MMP, corroborating the results we obtained with CmpdA treatment.

We next evaluated how siRNA-mediated IKKβ targeting would affect cell migration and invasion. IKKβ targeting reduced A549 cell migration and invasion by 38% and 50%, respectively (Figure 6C,D). This result was phenocopied by siRNA-mediated KRAS targeting, which reduced A549 migration and invasion by 46% and 41%, respectively (Figure 6C,D). Even though H358 cells do not display a migratory phenotype, both siRNA-mediated IKKβ and KRAS targeting reduced the invasive phenotype of these cells by 66% and 69%, respectively (Figure 6E). Similar to what we observed for tumoursphere formation, even though we cannot rule out the possibility that the reduction in H358 cell invasion upon KRAS silencing may reflect a loss of intrinsic viability caused by KRAS suppression, the reduction in migration and invasion observed in A549 cells and upon IKKβ inhibition cannot be attributed to an intrinsic cell viability loss. Taken together, these results demonstrate that IKKβ promotes KRAS-mutant lung cell migration and invasion potentially by promoting expression and activity of MMP2.

In conclusion, our results show that IKKβ promotes KRAS-induced stem-like and malignant traits, which indicates that IKKβ is likely involved in promoting tumour recurrence and aggressiveness, thereby suggesting IKKβ inhibition as a relevant therapeutic approach for KRAS-induced lung cancer.

## 3. Discussion

Tumour-initiating cells (TICs) represent a subpopulation of highly tumourigenic cancer cells with stem-like properties that are directly involved with therapy resistance, metastasis and recurrence [6]. The identification and targeting of these cells remain challenging due to their plastic behaviour, as well as the lack of knowledge on the molecular mechanisms that distinguish them from the other cells of the tumour bulk. Here, we present evidence that KRAS-driven TICs have increased IKKβ kinase activity, which, not only promotes stemness, but is also involved in tumour cell migration and invasion.

These findings are novel and relevant in various ways. First, accumulating evidence demonstrates that KRAS, one of the most frequently mutated genes in human cancers, plays a critical role in the maintenance of a cancer stem-like phenotype. KRAS is not only able to impair the differentiation of endodermal progenitors in vitro when exposed to retinoic acid [31], but also promotes maintenance and expansion of TICs in breast cancer [12,32], keratinocytes [33], prostate cancer [16], colon cancer [34], pancreatic cancer [14] and lung cancer [15].

Second, since the acquisition of stemness behaviour and malignant traits are linked through the epithelial-mesenchymal transition (EMT) program [35], it is not surprising that, in addition to promoting stemness, KRAS has also been implicated in promoting EMT, invasion and metastasis [9,11,36]. In fact, KRAS not only promotes EMT [10] and autophagy-dependent invasion [36], but also drives colorectal metastasis formation [11].

Finally, in spite of this evidence, the pathways triggered by KRAS to promote stemness, invasion and metastasis are only now beginning to emerge [14,15,34], most of which can be related to the IKK/NF-κB pathway. For example, KRAS has been shown to activate NF-κB in a ras-like proto-oncogene B (RalB)-dependent manner in order to promote stemness and drug resistance [13]. Additionally, IKKβ is a downstream target of protein kinase C iota (PKCι) [37], and PKCι targeting in KRAS-driven TICs greatly impairs tumourigenic potential in immunocompromised mice, tumoursphere formation and the expression of stemness-related genes [15]. Moreover, KRAS binds calmodulin and interfering with calmodulin binding reduces phosphatidylinositol 3-kinase PI3K activity and PI3K-mediated EMT, invasion and metastasis [38]. Interestingly, PI3K can activate the IKK/NF-κB pathway [39]. In accordance with the pathways uncovered by these reports, we show that KRAS-mediated maintenance of lung TIC activity and cell migration and invasion depends on IKKβ and, thereby, we identify IKKβ as a new druggable KRAS target involved in promoting a stem-like and invasive phenotype.

Consistent with its role as a canonical activator of NF-κB [19], which is activated in TICs from various cancers [20], and also promotes EMT and induces TIC activity in lung cancer [40], recent studies have shown that IKKβ can also promote cancer stemness. IKKβ targeting reduces breast cancer mammosphere formation and tumourigenicity [41], and positively regulates Lin28B and SRY (sex determining region Y)-box 2 (SOX2) to promote breast cancer stemness [42] and reduces breast GD2+ TICs, thereby reducing metastasis [43]. Consistently, an IKKβ inhibitor was identified as a hit in a screening of breast TIC inhibitors [44]. In addition, IKKβ targeting reduces prostate cancer tumoursphere formation and expression of stem cell factors [45], as well as reduces the ability of CD133+ glioblastoma TICs to form tumourspheres [46]. Moreover, IKKβ upregulates the long noncoding RNA HOX transcript antisense RNA (HOTAIR) to promote liver cancer stem cell growth in vitro and in vivo [47]. Finally, another study found that BMS-345541, an allosteric site-binding inhibitor of IKKβ, decreases tumoursphere formation and the expression of stem cell transcription factors of CD166+/CD44+ and CD166+/epithelial cell adhesion molecule (Epcam)+ A549 cells [48]. Here, we show, not only that IKKβ activity is increased in KRAS-mutant lung tumourspheres (Figure 1), but also that IKKβ targeting in KRAS-mutant lung cancer cells reduces expression of stem cell factors (Figure 2), as well as KRAS-mutant lung tumoursphere formation and self-renewal (Figure 3 and Figure 4). We also show that A549 tumoursphere-derived cells are preferentially sensitive to CmpdA (Figure 3), suggesting IKKβ inhibition as an interesting approach to selectively target KRAS-mutant TICs.

In addition to its role in promoting KRAS-induced stemness, we found that IKKβ also promotes KRAS-induced migration and invasion (Figure 5 and Figure 6). This is supported, not only by the well-established role of the NF-κB pathway in promoting invasion and metastasis [49], but also by studies showing that IKKβ is involved in promoting malignant behaviour. For example, in breast cancer, IKKβ-dependent NF-κB activation is essential to drive EMT and metastatic dissemination [50]. Furthermore, IKKβ targeting reduces EMT and metastasis in a colorectal mouse model of tumour progression [51] and reduces oral squamous carcinoma and prostate cancer cell invasion [52]. Finally, angiogenesis contributes to metastatic dissemination by shared mediators, such as the interleukin 8 (IL-8) cytokine [25,53], and IKKβ has been shown to upregulate IL-8 to promote lung cancer angiogenesis and ovarian cancer angiogenesis and metastasis [24,54].

Because invasion and metastasis are frequently associated with altered expression and activity of matrix metalloproteinases 2 (MMP2) and 9 (MMP9) [55], and because both have been shown to be regulated by NF-κB [19], we investigated how IKKβ and KRAS targeting affected the expression and activity of MMP2 and MMP9 (Figure 6). Even though IKKβ targeting reduced the expression of MMP9 as expected, surprisingly KRAS targeting only significantly decreased the expression of MMP9 in H358 cells. This cell type-specific regulation of MMP9 expression by KRAS could stem from the fact that, whereas A549 cells are homozygous for KRAS^G12S^, H358 cells are heterozygous for KRAS^G12C^. Not only different KRAS mutant forms have been shown to result in preferential activation of different signalling pathways, but wild type KRAS has been shown to have an inhibitory effect on the oncogenic KRAS form in cells that are heterozygous for KRAS mutations [56]. Nonetheless, MMP9 expression regulation by the KRAS/IKKβ pathway in these cells is likely unimportant, as targeting either KRAS or IKKβ in both lung cancer cell lines did not affect MMP9 enzymatic activity. On the other hand, IKKβ or KRAS targeting reduced both the expression and activity of MMP2, thus suggesting that KRAS-induced invasion is mediated, at least in part, by IKKβ-induced MMP2 expression. In support of this idea, KRAS has been shown to promote MMP2 expression and activity [57].

Interestingly, a recently published report claims that IKKα, the other catalytic subunit of the IKK complex, would be a better actionable target in KRAS-induced lung cancer, suggesting combined IKKα and IKKβ inhibition as a therapeutic approach [58]. It is possible that IKKβ targeting would affect IKKα activity, at least in part, as both associate in the IKK complex, but IKKα homodimers also associate in a second IKK complex that acts independently of IKKβ [19]. Nonetheless, IKKα’s role in KRAS-induced lung cancer remains controversial, as a second report claims that IKKα acts as a tumour suppressor and that IKKα genetic deletion actually promotes KRAS-induced lung cancer [59]. It is interesting and reassuring that these recently published antagonistic reports do not rule out IKKβ as a relevant target in KRAS-induced lung cancer. Our data gives support to the relevance of IKKβ as a therapeutic target by providing insight about the mechanism whereby IKKβ promotes KRAS-induced tumorigenesis.

Taken together, our results identify IKKβ as an important mediator of KRAS-induced stemness and invasion in lung cancer and indicate that IKKβ inhibition might selectively target cells with stem cell and invasive traits. This is important, not only because it further underscores the relevance of IKKβ as a therapeutic target for KRAS-induced lung cancer, but also because it suggests that using IKKβ inhibition therapy as an adjuvant approach to standard chemotherapy might constitute a promising strategy to lower recurrence rates, reduce metastatic dissemination and improve survival of KRAS-induced lung cancer patients.

## 4. Materials and Methods

### 4.1. Cell Lines and Culture Conditions

Human lung cancer cell lines harbouring KRAS mutations A549 (KRAS^G12S^) and H358 (KRAS^G12C^) were obtained from the American Type Culture Collection (ATCC; Manassas, VA,USA) and authenticated by short tandem repeat profiling at ATCC. Cells were grown in RPMI 1640 (Thermo Fisher Scientific, Waltham, MA, USA) with 10% foetal bovine serum (FBS, Sigma-Aldrich, St. Louis, MO, USA) in a humidified incubator at 37 °C and 5% CO_2_. Cells were treated with IKKβ inhibitor Compound A (CmpdA) [27] (kindly provided by Albert Baldwin) or 0.1% dimethyl sulfoxide (DMSO, vehicle control) as indicated in the figure legends.

### 4.2. Tumoursphere Formation

To generate TIC-enriched tumourspheres, 1 × 10^3^ cells (for counting and replating experiments) or 5 × 10^3^ cells (for protein and RNA analysis) were seeded in 6-well ultra-low attachment plates (Corning Inc., New York, NY, USA) and cultured in serum-free medium DMEM/F12 (Gibco, Carlsbad, CA, USA) supplemented with 20 ng/mL FGF (PeproTech, Rocky Hill, NJ, USA), 20 ng/mL EGF (PeproTech) and 1X N2 supplement (Gibco) for 14 days. Every 3 days, each well was supplemented with 500 µL of the same medium. After 14 days, tumoursphere number was determined by manual counting. Alternatively, tumourspheres were collected for protein or RNA analysis or for replating experiments. For replating assays, tumourspheres were dissociated with StemPro^®^ Accutase (Thermo Fisher Scientific) and reseeded for secondary and tertiary tumoursphere cultures (1 × 10^3^ cells) or for clonogenic assays (as described below). Treatment with Compound A was performed as described in the figures only once during seeding and siRNA transfection was performed 72 h before seeding of primary tumoursphere cultures.

### 4.3. siRNA Transfections

A549 and H358 cells were seeded in 6-well-plates (1 × 10^5^ cells/well) 12 h before transfection. siRNA transfections were performed using 50 nM of either siRNA SMARTpools targeting KRAS or IKKβ or a non-targeting siRNA control (Dharmacon, Lafayette, CO, USA) according to the manufacturer’s instructions.

### 4.4. Clonogenic Assay

Adherent and tumoursphere-derived cells were seeded at 5 × 102 cells (A549) or 5 × 103 cells (H358) per plate in 60 mm or 6-well adherent plates in triplicate. Cells were allowed to form colonies for 2 weeks with medium changed twice a week. Colonies were stained with 0.5% crystal violet solution for 10 min and colony number and/or area was quantified using ImageJ software (version 1.51, National Institutes of Health, Bethesda, MD, USA).

### 4.5. Quantitative Real-Time Polymerase Chain Reaction (qPCR)

Total RNA was isolated using TRIzol reagent (Thermo Fisher Scientific) following the manufacturer’s protocol and cDNA synthesis was performed with 1 μg of total RNA using Superscript III reverse transcriptase (Thermo Fisher Scientific). Relative expression of *BMI1*, *CD24*, *CXCR4*, *SOX2*, *OCT4*, *NANOG*, *KRAS*, *IKKβ*, *MMP2,* and *MMP9* was analysed by real-time PCR performed in a StepOnePlus Real-Time PCR System (Applied Biosystems, Foster City, CA, USA) using SYBR^®^ Green Master Mix (Thermo Fisher Scientific). Relative quantitation was performed by the ΔΔ*C*t method using *β-ACTIN* or *GAPDH* as endogenous controls. Primer sequences for each gene are described in Table S1.

### 4.6. Western Blotting

Radioimmunoprecipitation Assay (RIPA)^Hi^ buffer (150 mM NaCl, 50 mM Tris–HCl pH 7.5, 1% NP-40, 0.5% sodium deoxycholic acid, 1% SDS) containing protease and phosphatase inhibitors (Complete protease/phosphatase inhibitor cocktails, Sigma-Aldrich) was used to prepare whole cell lysates. Protein concentration was determined using Bradford Reagent (BioRad, Hercules, CA, USA) and electrophoresis was conducted with 50 μg of protein per lane in 12% polyacrylamide minigels in running buffer (25 mM Tris, 190 mM glycine, and 0.1% SDS) at 120 V for 60–90 min, followed by transfer to nitrocellulose membranes (Merck Millipore, Burlington, MA, USA) with Towbin buffer (25 mM Tris, 192 mM glycine, 20% methanol) at 250 V for 2.5 h. Membranes were blocked with 5% milk solution in TBST (20 mM Tris, pH 7.5, 150 mM NaCl, 0.1% Tween- 20) for 1 h at room temperature. Finally, membranes were incubated with primary antibodies diluted in TBST containing 5% BSA and 0.1% NaN_3_ for 16 h at 4 °C, washed 3 times in TBST and incubated with Horseradish Peroxidase (HRP)-conjugated secondary antibodies diluted in TBST for 1 h at room temperature. Chemiluminescence detection was performed using Pierce Enhanced Chemiluminescent (ECL) Western Blotting Substrate (Thermo Fisher Scientific) in a ChemiDoc MP Imaging System (BioRad). The following primary ant following primary ant ibodies were used: anti-phospho-IκBα^Ser32^ (1:1000, Cell Signalling, Danvers, MA, USA), anti-IκBα (1:1000, Cell Signalling), anti-β-Actin (1:7000, Sigma-Aldrich), anti-IKKβ (1:1000, Cell Signalling, Danvers, MA, USA), anti-PanRAS (1:1000, Merck Millipore), and anti-α-tubulin(1:2000, Sigma-Aldrich). The following secondary antibodies were used: HRP anti-rabbit (1:7000, GE Healthcare, Chicago, IL, USA) and HRP anti-mouse (1:7000, GE Healthcare). Protein bands of interest were quantitated using ImageJ as indicated in the figure legends.

### 4.7. Cell Proliferation, Cell Death and Wound-Healing Assays

Cell proliferation, cell death and wound-healing assays were analysed by IncuCyte time-lapse video microscopy (Essen Bioscience, Hertfordshire, UK). For cell proliferation and cell death, 3 × 10^3^ cells (A549) or 9 × 10^3^ cells (H358) were plated in a 96-well clear-bottomed black tissue culture plate (BD Falcon). On the next day, cells were treated with CmpdA or vehicle control (0.1% DMSO) as indicated in the figure legends. Cell proliferation was monitored over 96 h by recording cell density every 3 h. Cell death measurements were performed after 72 h of drug treatment using the *IncuCyte^®^ Sytox Green Reagent* (1/1000) to label dead cells. To calculate the percentage of dead cells, total cell number was quantified by incubation with 0.5% Triton X-100 for 1 h to permeabilise all cells to the *Sytox* dye. For wound-healing assays, 2.5 × 10^4^ A549 cells were plated in an ImageLock 96-well plate (Essen Bioscience). Cells were grown to confluence and a wound was created using the WoundMaker apparatus (Essen Bioscience) according to manufacturer instructions. The wells were then washed twice with PBS and medium was replaced according to the experiment design (0.1% DMSO or increasing doses of CmpdA). Plates were imaged over 96 h and cell density in the wound was recorded every 2 h. Data were analysed using the IncuCyte Confluence version 1.5 software, which quantified cell surface area coverage as confluence values. IncuCyte experiments were performed in triplicate. Two independent experiments were performed with technical triplicates and a single representative growth curve is shown for each condition.

### 4.8. Transwell Migration Assays

Migration assays were performed as previously described [24]. Briefly, using 24-well transwell inserts with 8 μm pore membrane filters (Corning). For pharmacological studies, migration assays were performed 24 h after A549 cells were treated with the IKKβ inhibitor CmpdA. Alternatively, cells were transfected with siRNAs as described above and migration was analysed 72 h post-transfection. In both cases, for each well 5 × 10^4^ A549 cells were resuspended in 300 μL serum-free medium, added to the upper chamber and incubated for 24 h at 37 °C in 5% CO_2_. Complete medium (500 μL) was added to the lower chamber to be used as chemoattractant. Non-migrating cells were scraped off the upper surface of the membrane with a cotton swab, and migrating cells on the bottom surface were fixed in 4% paraformaldehyde in PBS and stained with crystal violet. Images were obtained under an IX51 Inverted Microscope (Olympus, Tokyo, Japan) and cells from three random fields of view from three independent experiments were analysed using ImageJ software.

### 4.9. Invasion Assay

Invasion assays were performed using Matrigel-coated 24-well transwell inserts with 8 μm pore membrane filter (Corning Inc.). For pharmacological studies, invasion assays were performed 24 h after A549 cells and H358 cells were treated with the IKKβ inhibitor CmpdA. Alternatively, A549 cells and H358 cells were transfected with siRNAs and invasion was analysed 72 h post-transfection. In both cases, A549 (3 × 10^4^ cells per well) or H358 cells (6 × 10^4^ cells per well) were resuspended in 300 μL serum-free medium, added to the upper chamber and incubated for 24 h (A549 cells) or 48 h (H358 cells) at 37 °C in 5% CO_2_. Complete medium (500 μL) was added to the lower chamber to be used as chemoattractant. Non-invading cells were scraped off the upper surface of the membrane with a cotton swab, and invading cells on the bottom surface were fixed in 4% paraformaldehyde in PBS and stained with crystal violet. Images were obtained under an IX51 Inverted Microscope (Olympus). Cells from three random fields of view from three independent experiments were counted using ImageJ software.

### 4.10. Measurement of MMP2 and MMP9 Activity

A549 cells and H358 cells were transfected with a non-targeting control siRNA (siRNA) or with siRNA SMARTpool, targeting KRAS (siKRAS) or IKKβ (siIKKβ) and conditioned medium was collected 96 h post-transfection. Then, MMP2 and MMP9 activity levels were measured using specific Biotrak assay systems (MMP2 Biotrack Activity Assay RPN 2631 and MMP9 Biotrak Activity Assay RPN 2643, GE Healthcare) according to the manufacturer’s instructions. Each cell line was analysed in triplicate.

### 4.11. Statistical Analysis

Statistical analysis was performed using Prism 8 (GraphPad Software) as previously described [24]. All values are presented either as average ± SD or as representative images of at least two independent experiments. All data have been evaluated for normality of distribution. In order to assess significance in multiple comparisons, one-way analysis of variance (ANOVA) was used with a post hoc Turkey test. For pairwise comparisons, we used a non-parametric Student’s *t* test. Differences were considered statistically significant at *p* < 0.05.

### 4.12. Data Availability

The datasets generated during and/or analysed during the current study are available from the corresponding author on reasonable request.

## Supplementary Materials

Supplementary materials can be found at https://www.mdpi.com/1422-0067/21/16/5806/s1.

## Abbreviations

BMI1	B cell-specific Moloney murine leukaemia virus integration site 1
CmpdA	Compound A
CD24	Cluster of differentiation 24
CXCR-4	C-X-C chemokine receptor type 4
DMSO	Dimethyl sulfoxide
GAPDH	Glyceraldehyde 3-phosphate dehydrogenase
IKKβ	Inhibitor of nuclear factor kappa-B kinase β
IκB	Inhibitor of nuclear factor kappa-B
MMP2	Matrix metalloproteinase 2
MMP9	Matrix metalloproteinase 9
NF-κB	Nuclear factor-kappa-light-chain-enhancer of activated B cells
OCT4	Octamer-binding transcription factor 4
SOX2	SRY (sex determining region Y)-box 2
siRNA	Small interfering RNA
TICs	Tumour-initiating cells
