# Correction: Rodrigues et al. IKKβ Kinase Promotes Stemness, Migration, and Invasion in KRAS-Driven Lung Adenocarcinoma Cells. *Int. J. Mol. Sci.* 2020, *21*, 5806

**DOI:** 10.3390/ijms27188004

**Published:** 2026-09-09

**Authors:** Felipe Silva Rodrigues, Vanessa Silva Miranda, Tatiana Correa Carneiro-Lobo, Luiza Coimbra Scalabrini, Björn Kruspig, Elena Levantini, Daniel J. Murphy, Daniela Sanchez Bassères

**Affiliations:** 1 Departamento de Bioquímica, Instituto de Química, Universidade de São Paulo, São Paulo 05508-000, Brazil; 2 Institute of Cancer Sciences, University of Glasgow, Glasgow G61 1QH, UK; 3 Beth Israel Deaconess Medical Center, Harvard Medical School, Boston, MA 02115, USA; 4 Istituto di Tecnologie Biomediche, Consiglio Nazionale dele Ricerche, 56124 Pisa, Italy; 5 Cancer Research UK Beatson Institute, Glasgow G61 1BD, UK

**Error in Figure**

In the original publication [1], the Western blot images for phospho-IκBα, IκBα, and β-actin in Figure 2A were inadvertently duplicated from a separate publication. Because the experiments in both studies used identical cell lines, treatments, antibodies, and protocols, the same immunoblot image was mistakenly included during figure assembly. This panel serves to confirm that Compound A treatment inhibits IKKβ kinase activity, as demonstrated by us and others. The error occurred during figure preparation. The experiments were independently performed using identical protocols, and the results are consistent with those reported in the manuscript. A corrected Figure 2A, containing immunoblot images from the independent experiment, is provided below. 

The authors state that the scientific conclusions are unaffected. This correction was approved by the Academic Editor. The original publication has also been updated.

## Figures and Tables

**Figure 2:**
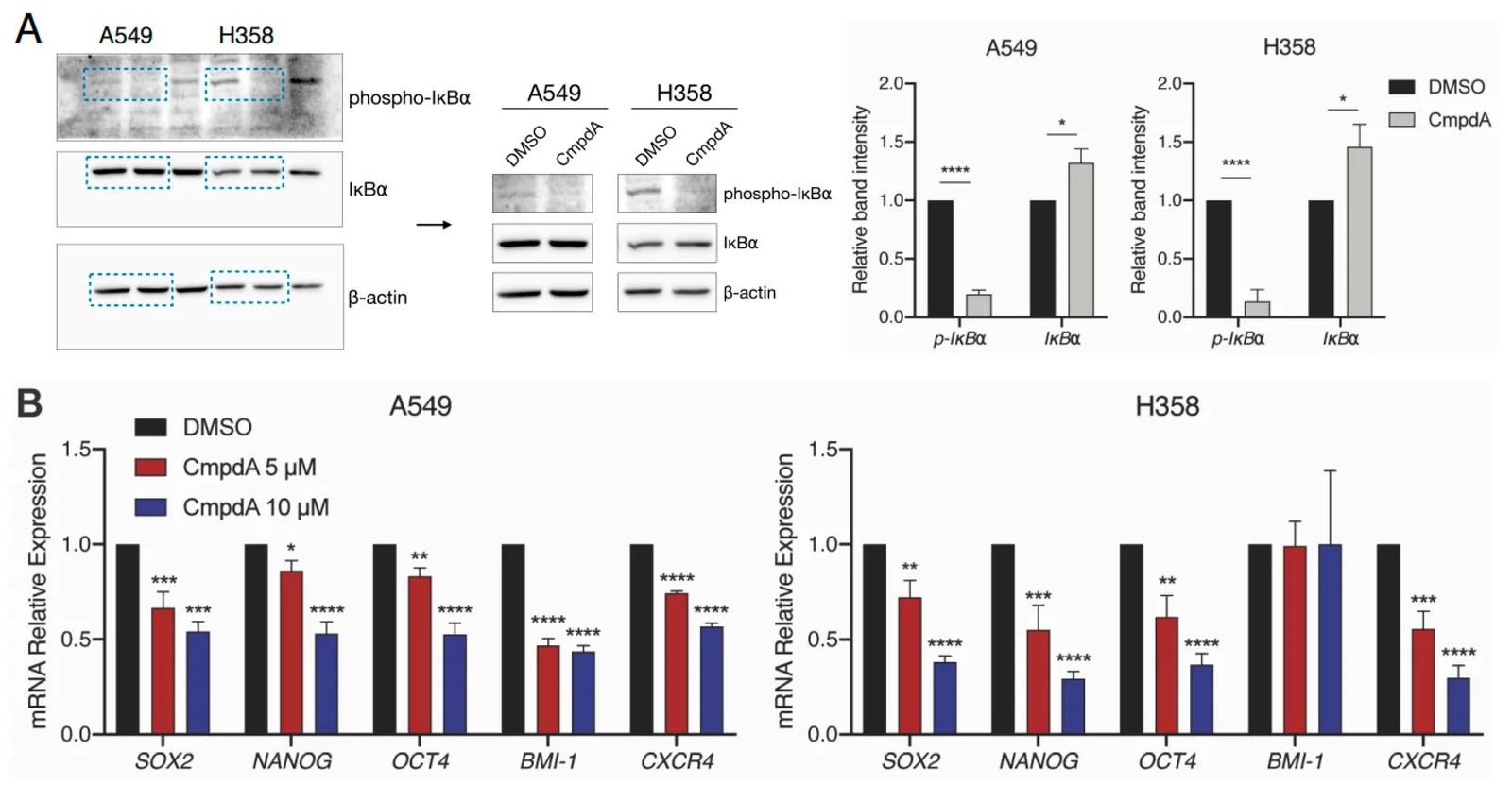
IKKβ targeting reduces the expression of stemness-associated genes in KRAS-mutant lung cancer cells. (**A**) Western blotting of A549 and H358 cells treated with 0.1% DMSO or 5 µM Compound A (CmpdA) for 30 min. Antibodies were used as indicated. Nitrocellulose membrane was cut before probing with the respective primary antibody, and full membrane blots are presented in Figure S2. The representative Western blots are shown. The protein bands were quantitated and normalized to the reference samples (0.1% DMSO-treated samples) using Image J software (version 1.51). (**B**) A549 and H358 cells were treated with 0.1% DMSO or the indicated concentrations of CmpdA for 48 h, and expression of *SOX2*, *OCT4*, *NANOG*, *BMI1*, and *CXCR4* was evaluated by qRT-PCR using *β-ACTIN* as an endogenous control. The bar graphs represent average ±1 SD of three independent experiments (n = 3). Statistical significance was determined by one-way ANOVA with the post hoc Turkey test. (* *p* < 0.5, ** *p* < 0.01, *** *p* < 0.001, **** *p* < 0.0001) by comparing the CmpdA-treated groups with the DMSO-treated group.
