# Supplementary material for: IKKβ Kinase Promotes Stemness, Migration, and Invasion in KRAS-Driven Lung Adenocarcinoma Cells
Source: Int J Mol Sci. 2020 Aug 13;21(16):5806. doi: 10.3390/ijms21165806 (PMC7460870; doi:10.3390/ijms21165806)
Supplement: Supplementary file 1 [file ijms-21-05806-s001.pdf]

Supplementary Figure S1.

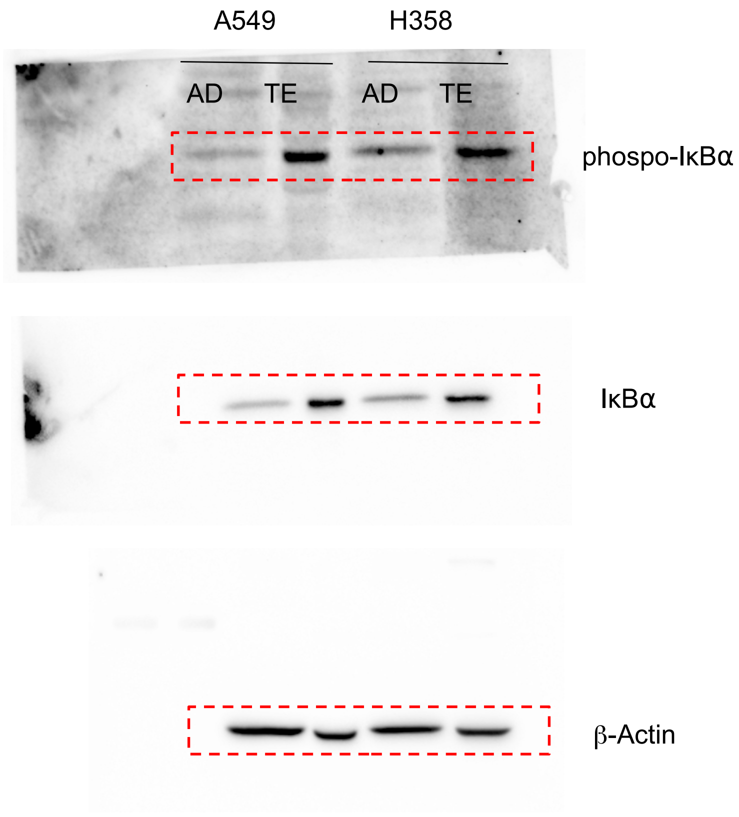

Supplementary Figure S1. Supporting blot data for Figure 1c. Western blotting of A549 and H358 cells growing in adherent or tumoursphere condition. Antibodies were used as indicated. Nitrocellulose membrane was cut before probing with the respective primary antibody and full membrane blots are shown. Cropped areas used in Fig. 1c are indicated by red-dashed rectangles.

Supplementary Figure S2.

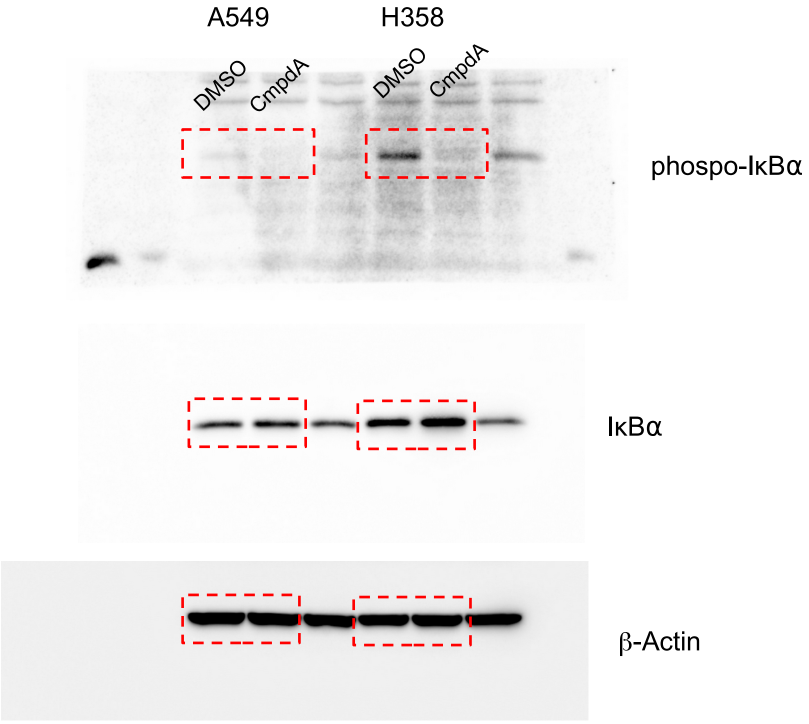

Supplementary Figure S2. Supporting blot data for Figure 2a. Western blotting of A549 and H358 cells treated with 0.1% DMSO or 5 μM CmpdA for 30 min. Antibodies were used as indicated. Nitrocellulose membrane was cut before probing with the respective primary antibody and full membrane blots are shown. Cropped areas used in Fig. 2a are indicated by red-dashed rectangles.

Supplementary Figure S3.

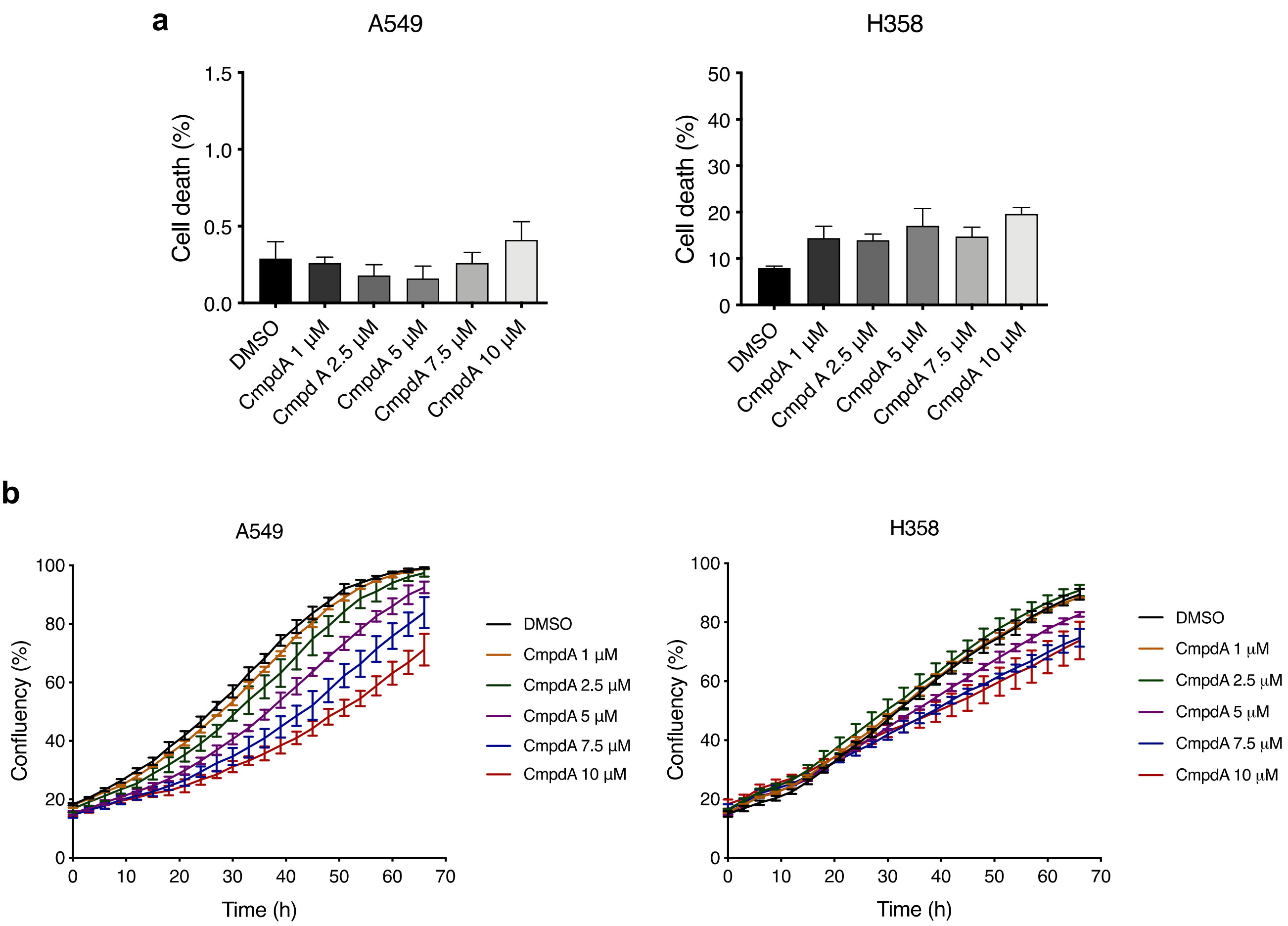

Supplementary Figure S3. IKKβ inhibition in A549 cells reduces proliferation in a dose-dependent manner without causing cell death. (a) A549 and H358 cells were treated with 0.1% DMSO or the indicated concentrations of CmpdA and cell proliferation and death were analysed by IncuCyte time-lapse video microscopy as described in methods. Bar graphs represent average ± 1 SD of two independent experiments. (b) Growth curves measured by IncuCyte time-lapse video microscopy of KRAS-mutant A549 cells (left) or H358 cells (right) upon treatment with 0.1% DMSO or the indicated concentrations of CmpdA. A representative growth curve of two independent experiments is shown for each condition. Error bars show ± 1 SD for technical triplicates.

Supplementary Table S1. Sequence of forward and reverse primers used for real-time PCR analysis.

Gene	Primers	Amplicon size	Gene bank accession number
ACTB (β-actin)	$SeqF_w$: 5'GGCACCCAGCACAATGAAG-3'<br>$SeqR_v$: 5'- CCGATCCACACGGAGTACTTG-3'	67 bp	NM_001101.5
BMI1	$SeqF_w$: 5'- CCCACCTGATGTGTGTGCT-3'<br>$SeqR_v$: 5'- GGTCTCCAGGTAACGAACAATACA-3'	107 bp	NM_005180.9
CD24	$SeqF_w$: 5'- GACATGGGCAGAGCAATGGTGGC-3'<br>$SeqR_v$: 5'- GAGTGAGACCACGAAGAGACTGGC-3'	222 bp	NM_001359084.1
CXCR4	$SeqF_w$: 5'- CCAGTAGCCACCGCATCT-3'<br>$SeqR_v$: 5'- GGTGGGCAGGAAGATTTTATTG-3'	165 bp	NM_003467.3
SOX2	$SeqF_w$: 5'- CCCGGCGGAAAACCAA-3'<br>$SeqR_v$: 5'- GCCGGGCAGCGTGTACT-3'	53 bp	NM_003106.4
POU5F1 (OCT4)	$SeqF_w$: 5'- TCGAGCAATTTGCCAAGCT-3'<br>$SeqR_v$: 5'- GCCTCAAAGCGGCAGATG-3'	130 bp	NM_001159542.2
NANOG	$SeqF_w$: 5'- CCTCAGCCTCCAGCAGATG-3'<br>$SeqR_v$: 5'- CAGTTGTTTTCTGCCACCTCTT-3'	116 bp	NM_001355281.2
KRAS	$SeqF_w$: 5'- CCCAGGTGCGGGAGAGA-3'<br>$SeqR_v$: 5'- CAGCTCCAACTACCACAAGTTT-3'	63 bp	NM_033360.4
IKBKB (IKKβ)	$SeqF_w$: 5'- GCCCTTCCTCCCCAACTG-3'<br>$SeqR_v$: 5'- TCTTCTGCCGCACTTTTGAA-3'	56 bp	NM_001190720.3
MMP2	$SeqF_w$: 5'- TGGCTACACACCTGATCTGG-3'<br>$SeqR_v$: 5'- GAGTCCGTCCTTACCGTCAA-3'	184 bp	NM_004530.6
MMP9	$SeqF_w$: 5'- TCTTCCCTGGAGACCTGAGA-3'<br>$SeqR_v$: 5'- TTTCGACTCTCCACGCATCT-3'	107 bp	NM_004994.3
GAPDH	$SeqF_w$: 5'-GAGCCGCATCTTCTTTTGC-3'<br>$SeqR_v$: 5'-CCATGGTGTCTGAGCGATGT-3'	54 bp	NM_001357943.2

Supplementary Figure S4.

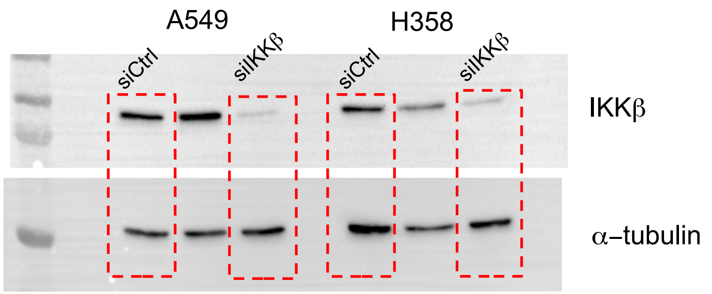

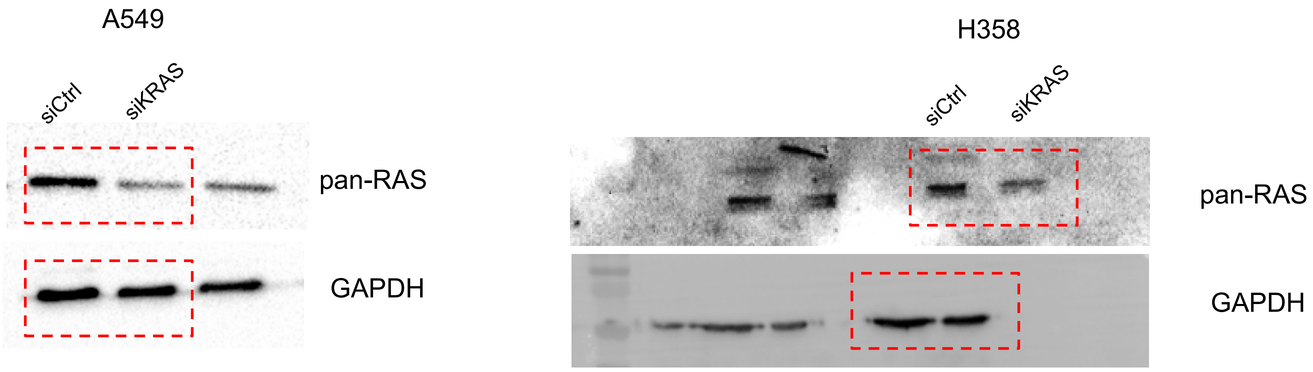

Supplementary Figure S4. Supporting blot data for Figure 4a. Western blotting of A549 and H358 cells treated with the indicated siRNA. Antibodies were used as indicated. Nitrocellulose membrane was cut before probing with the respective primary antibody and full membrane blots are shown. Cropped areas used in Fig. 4a are indicated by red-dashed rectangles.
